# Predictors of Quit Attempts and Successful Smoking Abstinence in Pregnancy: An Exploratory Analysis of a Pooled Clinical Trial Dataset

**DOI:** 10.1093/ntr/ntaf262

**Published:** 2025-12-24

**Authors:** Hannah Igoe, Joanne Emery, Felix Naughton, Jaspal Taggar, Jo Leonardi-Bee, Tim Coleman

**Affiliations:** Centre for Academic Primary Care, Lifespan and Population Health, School of Medicine, University of Nottingham, Nottingham, UK; School of Health Sciences, University of East Anglia, UK; School of Health Sciences, University of East Anglia, UK; Centre for Academic Primary Care, Lifespan and Population Health, School of Medicine, University of Nottingham, Nottingham, UK; Faculty of Medicine and Health Sciences, Lifespan and Population Health, University of Nottingham, UK; Centre for Academic Primary Care, Lifespan and Population Health, School of Medicine, University of Nottingham, Nottingham, UK

## Abstract

**Introduction:**

Smoking in pregnancy is a preventable cause of detrimental effects on mother and baby, exacerbating socioeconomic health disparities. This study investigates predictors of making a quit attempt and smoking abstinence in pregnancy.

**Methods:**

This study used pooled data from two large UK multicenter smoking cessation intervention trials in pregnancy (*N* = 1409). Baseline predictor variables included demographic, smoking behavior, and quitting belief measures. Percentage of cigarettes cut down in early pregnancy was a novel variable. Outcomes (36 weeks’ gestation) were having made a quit attempt (>24 hours) and smoking abstinence (both self-reported). Exploratory logistic regression analyses, with missing smoking outcomes imputed as non-abstinent, involved univariate then multivariable analyses. Sensitivity analyses included complete case analyses and biochemically validated abstinence.

**Results:**

A total of 1409 women were included in study analyses. In multivariable analyses, for making a quit attempt, higher intention to quit (OR = 1.50, 95% CI = 1.26% to 1.79%) and higher combined self-efficacy score (OR = 1.60, 95% CI = 1.30% to 1.96%) were statistically significant predictors. For smoking abstinence, higher percentage reduction in number of cigarettes smoked in early pregnancy (OR = 1.02, 95% CI = 1.01% to 1.02%) and longer previous quit attempt (omnibus *p* = .001) were statistically significant predictors.

**Conclusions:**

Smoking beliefs, including motivational factors, were statistically significant predictors of making a quit attempt. However, smoking behaviors relating to cutting down the number of cigarettes in early pregnancy and having had a previous period of smoking abstinence >6 weeks were predictors of successful smoking abstinence.

**Implications:**

This study adds to the evidence that motivational factors are important in initiating a quit attempt, whereas positive behavioral changes are associated with smoking abstinence in pregnant women. This information could be used by clinicians designing smoking abstinence support for pregnant women. Women who reduce the number of cigarettes they smoke very early in pregnancy might be more receptive to smoking abstinence support offered at that point.

## Introduction

Smoking in pregnancy is one of the main preventable causes of morbidity and mortality in pregnant women. For expectant mothers, the main risks of smoking in pregnancy are ectopic pregnancy, placental abruption, and placenta previa; along with stroke, peripheral vascular disease, and heart disease. For the developing fetus, smoking can cause serious negative outcomes including miscarriage,^1^ still birth,^2^ congenital abnormalities,^3^ prematurity, intrauterine growth restriction, and subsequent low birth weight.^4^ Children exposed to smoking in-utero, are at increased risk of neonatal death, sudden infant death,^5^ poor cognition,^6^ and behavioral issues.^7^

Currently, in the United Kingdom, 13.6% of women smoked in early pregnancy with 7.5% of women smoking throughout pregnancy,^8^^,^^9^ and around half of women who smoke attempt to stop when pregnant.^8^ However, only 25% of women who smoke in pregnancy have a period of smoking abstinence,^10^ and up to 66% of these women start smoking again in the postnatal period.^11^ The proportion of women who smoke in pregnancy is higher in younger women, first pregnancies, and White women.^9^ Rates are substantially higher in populations with more social deprivation^8^ and children born to mothers who smoked during pregnancy are more likely to smoke themselves (including in adolescence),^12^ therefore increasing their risk of risk of non-communicable disease.^13^ Therefore, generational healthcare inequalities are exacerbated by smoking in pregnancy and it is a major issue for population health.

This is an international issue, with the highest prevalence countries being Ireland, Uruguay, and Bulgaria; and with a prevalence of 5.9% across Europe.^10^ Smoking cessation for pregnant women could reduce the risk of adverse pregnancy events and improve birth outcomes, for example birth weight.^14^ Also, compared with women who smoked before and during pregnancy, women who reduced or quit were twice as likely to be a non-smoker at age 55 years,^15^ therefore potentially improving healthcare outcomes beyond the pregnancy period.

It is important to identify factors that influence the quitting process, which theoretically has different stages.^16^ At its simplest form, quitting smoking involves making a quit attempt (initiation) and remaining abstinent for a specific duration (maintenance). By identifying modifiable factors at each of these two stages, we can then try to target these with interventions and so improve women’s chances of quitting (eg, by boosting their motivation). Identifying non-modifiable factors (eg, demographics) can help us know where to target support.

In a systematic review describing the significant factors associated with smoking cessation in pregnancy, the most frequently observed predictors were higher socio-economic status, cohabitation, primiparity, lower exposure to passive smoking, good mental health, not drinking alcohol, planned breastfeeding, lower nicotine dependence, and higher self-efficacy.^17^ Other predictors discussed in the literature include: planned pregnancy,^18^ higher motivation, smoking fewer cigarettes per day,^19^ lower stress, no previous use of marijuana,^20^ and having earlier prenatal care.^21^

Most previous studies have not distinguished between factors influencing women who attempt to quit smoking and those who become abstinent. This is potentially important as the process of initiating and maintaining abstinence is likely to have different influences. A study by Emery et al. recognized that the factors associated with making a quit attempt and successful abstinence were different and found that smoking beliefs predicted trying to quit among pregnant women, but not success in abstinence, whereas nicotine dependence was inversely associated with quitting but not with making a quit attempt.^22^ However, the study was limited by a small sample size, restricting power to detect associations, and therefore a larger study is warranted. We report an analysis using a substantially larger dataset than Emery et al., with the aim of potentially detecting associations not identified previously.

This study aims to determine the predictors of: (1) making a quit attempt, and (2) of smoking abstinence among pregnant women who smoked and were recruited to two multicenter UK trials.^23^^,^^24^

## Methods

### Design and Randomization

A pooled dataset (*n* = 1409) of pregnant smokers enrolled into two trials—the MiQuit Pilot^23^ (*n* = 407) and MiQuit3^24^ (*n* = 1002) was used in the analysis. These were multicenter parallel group RCTs with identical procedures and outcomes, which tested a 12-week tailored text message-based smoking cessation program. Randomization used a 1:1 ratio and was stratified by gestation (and site in MiQuit Pilot) at baseline (>16 weeks or <16 weeks). A statistical analysis plan was registered prior to statistical analyses being carried out for this exploratory cohort study.^25^

### Participants

Participants were from 40 National Health Service (NHS) hospital antenatal clinics in England, recruited between February and September 2014 (MiQuit Pilot), or December 2017 and February 2019 (MiQuit3). The inclusion criteria for both trials were age >16 years; <25 weeks’ gestation (at baseline); smoking >1 daily cigarette (5 prior to pregnancy); able to receive and understand texts in English. Participants were excluded from analysis if they experienced miscarriage or stillbirth prior to follow-up. Self-report questionnaires were completed at baseline (mean 15 weeks’ gestation), 4 weeks after randomization, and in late pregnancy around 36 weeks’ gestation. For participants that reported 7-day smoking abstinence at 36-week follow-up, saliva samples were requested for biochemical validation to measure cotinine levels. Full details of both RCTs are published elsewhere.^23^^,^^24^

### Predictor Measures

All predictor variables were measured at baseline. The theoretical underpinning for considering these measures is described in the Supplementary Material of the MiQuit Pilot trial outcomes paper.^23^

#### Demographic/Background Information

Demographic/background variables included Index of Multiple Deprivation Score,^26^ age, partner smoking status (partner non-smoker/no partner/partner smoker), and whether the participant had experienced a previous pregnancy (yes/no). Index of Multiple Deprivation score was based on 2015 neighborhood data.^26^

#### Smoking Behavior Variables

Baseline smoking behavior variables included the following: length of longest quit attempt prior to baseline (<2 weeks; 2–5 weeks; 6–11 weeks; >12 weeks), whether a date for quitting smoking was set (yes; no), intention to quit smoking (within 2 weeks; 30 days; 3 months; not seriously intending to quit). Baseline nicotine dependence and percentage change in number of cigarettes smoked in early pregnancy were continuous variables. Nicotine dependence was determined using the Heaviness of Smoking Index (HSI)^27^ and the strength of smoking urges in the past 24 hours (no urges; slight; moderate; strong; very strong; extremely strong).^28^ The percentage change in number of cigarettes smoked in early pregnancy was calculated using the difference between the number of daily cigarettes the participant reported smoking at baseline compared to before pregnancy (both measured at baseline, around 15 weeks), expressed as a percentage of the pre-pregnancy rate.

#### Smoking-Related Beliefs Variables

Baseline smoking belief variables were measured on a 5-point scale (not at all; a little; moderately; very much and extremely): determination to stop smoking for remainder of pregnancy, and beliefs regarding smoking causing harm to baby. Combined self-efficacy score was calculated using the average of four items measuring confidence to avoid smoking: one for the remainder of the pregnancy, and three in different types of tempting situation (after a meal; with other smokers; and when anxious/stressed). Each item is scored on a 5-point scale (“not at all” = 1 to “extremely” = 5).^23^

### Outcome Measures

In the MiQuit trials, smoking outcomes measured at 36 weeks’ gestation included: (1) having made at least one self-reported quit attempt lasting at least 24 hours; (2) self-reported point prevalence of 7-day abstinence from smoking; (3) biochemically validated smoking abstinence (via salivary cotinine or anabasine, or carbon monoxide breath test).^29^ Due to higher percentage of data completeness, we used the self-reported abstinence measure as the main outcome and conducted a sensitivity analysis using the validated outcome.

### Data Analysis and Attrition

Prior to merging of the data from the two trials, analysis was undertaken to ensure that the baseline characteristics were sufficiently similar. Potential group differences between MiQuit Pilot^23^ and MiQuit3^24^ baseline variables were tested using two-sided *t* test for normally distributed data and Mann–Whitney U test for non-normally distributed data.

To assess whether completeness of follow up might influence analysis outcomes, we compared baseline variables for participants by lost to follow-up status.

Exploratory logistic regression analyses were undertaken to identify which predictor variables were significantly associated with the two outcome measures. Initially, univariable analyses were conducted to examine the association between each of the predictor variables and each outcome. Assessments of collinearity between the predictor variables were performed using Pearson’s correlation coefficient. Participants were excluded from the analyses where there was missing predictor data; this equated to less than 5% of participants (*n* = 69).

Multivariable analyses were then built and augmented to examine the effect of each predictor variable when adjusting for all other predictor variables. All variables identified as significant (*p*<.05) in the univariable analyses were added to the regression model. The variables which became non-significant in this model were removed. Variables that were not significant in the univariable analysis were then added one at a time into the regression model to examine whether they became significant. This resulted in a final parsimonious model for each outcome variable, which identified predictor variables that were statistically significant when controlling for all the other predictor variables. Variables relating to allocation to treatment (MiQuit or control) and trial identification (MiQuit Pilot or MiQuit3) were included in the multivariable models. Regression diagnostics (Cook’s distance and leverage) were also performed. Results from the models are presented as odds ratios (ORs) with 95% confidence intervals (CIs).

Analyses for the abstinence outcome were restricted to the participants who made a quit attempt. For those with missing smoking outcome data, it was assumed that those who did not provide information were still smoking in line with the widely accepted Russell Standard, which is considered a more conservative approach than complete case analysis.^30^

As the majority of women in the trial made a quit attempt, this was considered to be the likeliest behavioral outcome for those with missing quit attempt outcomes, and it was assumed that those with missing outcome data had made a quit attempt, which is consistent with a previous similar study.^22^ Sensitivity analyses using complete case analysis were undertaken to assess whether these assumptions impacted the findings from the analyses. Further sensitivity analyses were performed using the biochemically validated abstinence outcome and also stratifying by trial condition.

Analyses were conducted using Statistical Package for the Social Sciences (SPSS) and Stata. Wald and Likelihood ratio test *p* values < .05 were used to indicate statistical significance.

## Results

### Participant Characteristics

We proceeded with analyses using both studies’ data combined because any differences in the baseline characteristics between the two studies were unlikely to be clinically significant or capable of influencing analysis finding, given that there were no notable differences in the methodology of the two studies (Appendix Table S1). This dataset contained data for 1409 trial participants (407 from MiQuit Pilot, 1002 from MiQuit3) and there was follow up (outcome) data for 906 (49.9%) of participants; 64.1% (261/407) of MiQuit Pilot participants and 64.5% (646/1002) of the MiQuit3 participants were present at follow up.

Table 1, for the merged trials’ dataset, shows the participant numbers and characteristics at baseline (*n* = 1409) and follow-up (*n* = 906) and compares the characteristics of both. There was a single statistically significant difference where those present at follow-up were more likely to have been pregnant previously, compared to all those present at baseline (*p* = .036).

**Table 1:** Baseline Characteristics of Participants Present Versus Lost at Follow-up in Merged Trials’ Dataset

Characteristic	Provided outcome data		Did not provide outcome data		
	*N* = 906^1^	%	*N* = 503^1^	%	*p* ^2^
**Trial Details**					
**Allocation**					
Intervention	437	48.2%	267	53.1%	.081^3^
Usual care	469	51.8%	236	46.9%	
**Demographics**					
**IMD** (*median, interquartile range*)^4^	6786.0, 11 085		6136.0, 11 214		.120^5^
*N, missing*	*889, 17*		*499, 4*		
**Age**					
Age (*mean, standard deviation)*	27.21, 5.622		26.82, 5.735		.209^6^
**Has a partner who smokes**					
Partner smokes	574	63.4%	332	66.0%	.528^3^
Partner doesn’t smoke	176	19.4%	95	18.9%	
No partner	156	17.2%	76	15.1%	
**Ethnicity** ^7^					
White	838	92.9%	480	95.6%	.051^3^
Mixed race	35	3.9%	16	3.2%	
Other	29	3.2%	6	1.2%	
*N, missing data*	*902, 4*		*502, 1*		
**Smoking Behaviors**					
**Percentage change in number of cigarettes smoked in early pregnancy**^8^					
Percentage change in number of cigarettes smoked in early pregnancy (m*edian, interquartile range)*	50.0, 33		50.0, 40		.095^5^
**Nicotine dependence (Using HSI score^9^)**					
Nicotine dependence (Using HSI) (m*ean, standard deviation)*	1.93, 1364		2.01, 1.378		.267^6^
**Longest quit attempt prior to baseline**					
Not attempted	207	22.8%	126	25.0%	.399^3^
<2 weeks	193	21.3%	109	21.7%	
2–5 weeks	131	14.5%	70	13.9%	
6–11 weeks	75	8.3%	28	5.6%	
>12 weeks	300	33.1%	170	33.8%	
**Previous pregnancy (proxy for smoking in previous pregnancy)^10^**					
Yes	586	64.7%	353	70.2%	.036^*3^
No	320	35.3%	150	29.8%	
**Strength of smoking urges (scale 1 = no urges; 6 = extremely strong)**					
Strength of smoking urges *(mean, standard deviation)*	3.13, 1.001		3.10, 1.046		.631^6^
*N, missing data*	*886, 20*		*484, 19*		
**Intention to quit smoking**					
Within 2 weeks	241	26.7%	144	28.6%	.527^3^
Within 30 days	237	26.2%	135	26.8%	
Within 3 months	337	37.3%	186	37.0%	
No intention to quit	88	9.7%	38	7.6%	
*N, missing data*	*903, 3*		*503, 0*		
**Set a quit date**					
Yes	55	6.1%	29	5.8%	.817^3^
No	851	93.9%	474	94.2%	
**Smoking beliefs^11^**					
Determination to stop smoking for remainder of pregnancy (*median, interquartile range)*	4.00, 2		4.00, 1		.400^5^
Combined self-efficacy^12^ (*mean, standard deviation*)	2.68, 0.818		2.73, 0.871		.262^6^
Beliefs regarding harm to baby (*median, interquartile range*)	5.00, 1		5.00, 1		.596^5^
*N, missing data*	*905, 1*		*503, 0*		

^1^ *N* = 906 for present at follow-up, *N* = 503 for lost at follow-up, unless otherwise stated.

^2^Tested using chi-squared test (frequencies) or *t* test (means), for normally distributed or Mann–Whitney U test (medians) for non-parametric data; two-tailed.

^3^Chi-squared test.

^4^Index of multiple deprivation rank (based on postcode based on 2015 data for both MiQuit3 and MiQuit Pilot). MD Rank in England covers domains of income, employment, health, education, crime, access to services, and living environment.^44^

^5^Mann–Whitney U test.

^6^ *t* test.

^7^Ethnicities with percentage >1% will be included in the table, and in those ethnicities where there is less than 1% of the participants in that category, will be grouped as “other”.

^8^Percentage change in cigarettes smoked in early pregnancy was calculated using the reported number of cigarettes smoked prior to pregnancy versus reported smoked at baseline.

^9^Using Heaviness of Smoking Index (HSI) score; combining the score of two items: cigarettes per day (1–5 = score of 0, 6–10 = 1, 11–20 = 2, 21–30 = 3, >30 = 4) and time to first cigarette after waking (>2 h = 0, 1–2 h = 1, 31–59 min = 2, ≤30 min = 3). A combined score of 0–2 = low dependence, 3–4 = medium dependence, 5–7 = high dependence.

^10^Previous studies have shown that smoking in a previous pregnancy and having had a previous pregnancy are very closely related among current pregnant smokers.^45^

^11^Smoking beliefs questions were answered using a scale where 1—not at all; 2—a little; 3—moderately; 4—very much; 5—extremely.

^12^The average of four items measuring confidence to avoid smoking: one for the remainder of the pregnancy, and three in different types of tempting situation (after a meal; with other smokers; and when anxious/stressed), as used in previous studies (α = 0.81).^23^  ^*^p < 0.05.

### Outcome Event Rates

Seventy-five percent (681/ 906) of participants present at follow-up reported a quit attempt. This includes nine who did not report making a quit attempt but who did report abstinence, and who, consistent with other studies, were reclassified as having made a quit attempt.^31^ Twenty two percent (199/906) reported 7-day smoking abstinence at the end of pregnancy, and 46% (91/199) had abstinence biochemically validated (108 failed the validation or failed to provide a sample).

One hundred ninety-nine women self-reported abstinence and, of these, 68 didn’t provide a saliva or carbon monoxide (CO) sample and 131 did. Of those who provided a sample, 91 passed and 40 failed. In summary, 108/199 failed validation.

### Predictors of Making a Quit Attempt

Table 2 shows the findings from the univariate and multivariable logistic regression analyses. Collinearity checks between predictor variables all had a Pearson correlation coefficient of *r* < 0.8, and regression diagnostics were satisfactory.

**Table 2:** Univariate and Multivariate Predictors of Making a Quit Attempt and Predictors of Self-reported Abstinence in Pregnant Smokers

		Quit attempt^1^		Self-reported abstinence^2^	
Predictor variable		Univariate OR^3^ (95% CI) *p* value^4^^*^	Multivariate OR (95% CI) *p* value^*^	Univariate OR (95% CI) p Value^*^	Multivariate OR (95% CI) *p* value^*^
Condition (0 = usual care, 1 = MiQuit)		1.535 (1.135 to 2.075)^*^ .005^*^	1.527 (1.120 to 2.082)^*^ .008^*^	1.246 (0.917 to 1.693) .160	
IMD Score (continuous, 15–32 742, increasing IMD)		1.000 (1.000 to 1.000) .237		1.000 (1.000 to 1.000) .475	
Age (continuous, 16–43, increasing age)		0.982 (0.957 to 1.008) .169		1.017 (0.990 to 1.044) .221	
Partner smoking status (Reference = Partner non-smoker)	Overall likelihood ratio (LR)	LR Chisquared = 3.223 DF = 3 *p*=.200 *(In STATA LR Chi*^2^ *= 3.22; p=.1996)*		LR Chi squared 2.698 DF = 2 *p*=.259	
	Category				
	*Single*	0.678 (0.401 to 1.138) .095		0.785 (0.476 to 1.280) .335	
	*Partner smoker*	0.699 (0.452 to 1.050) .881		0.729 (0.505 to 1.064) .096	
Percentage change number of cigarettes early pregnancy (Continuous, −300 to 97%, increasing percentage cut down)		1.009 (1.005 to 1.013)^*^ <.001^*^		1.016 (1.010 to 1.023)^*^ <.001^*^	1.015 (1.009 to 1.022)^*^ .001^*^
Nicotine dependence score (continuous, increasing HSI)		0.794 (0.711 to 0.887)^*^ <.001^*^		0.834 (0.745 to 0.935)^*^ .002^*^	
Previous quit attempt (Reference = Quit not attempted)	Overall LR	LR Chi^2^= 10.70 *p*=.030^*^		LR Chi^2^= 20.987 *p* = <.001	LR Chi^2^= 17.99 *p*=.001^*^
	Category				
	*<2 weeks*	0.832 (0.551 to 1.254) .379		1.032 (0.597 to 1.777) .909	1.084 (0.627 to 1.874) .773
	*2–5 weeks*	1.752 (1.030 to 3.087) .044		1.589 (0.923 to 2.735) .093	1.568 (0.908 to 2.707) .106
	*6–11 weeks*	1.230 (0.669 to 2.399) .522		2.028 (1.056 – 3.808) .030^*^	1.949 (1.023 to 3.716)^*^ .043^*^
	*>12 weeks*	1.375 (0.920 to 2.053) .119		2.268 (1.476 to 3.567)^*^ <.001^*^	2.208 (1.416 to 3.444)^*^ <.001^*^
Previous pregnancy (0 = No previous pregnancy; 1 = Had previous pregnancy)		0.793 (0.572 to 1.097) .162		0.759 (0.555 to 1.039) .085	
Strength of urges (continuous, 1–6, increasing strength)		0.952 (0.823 to 1.102) .509		0.941 (0.807 to 1.097) .439	
Intention to quit (continuous, 1–4, increasing intention)		1.726 (1.463 to 2.036)^*^ <.001^*^	1.501 (1.261 to 1.788)^*^ <.001^*^	1.369 (1.159 to 1.616)^*^ <.001^*^	
Set a quit date (0 = no, 1 = yes)		4.800 (1.502 to 15.344)^*^ .008^*^		2.457 (1.492 to 4.044) <.001^*^	
Determination to stop (continuous, 1–5, increasing determination)		1.623 (1.399 to 1.882)^*^ .001^*^		1.471 (1.212 to 1.786)^*^ <.001^*^	
Combined self-efficacy (continuous, 1–5, increasing self-efficacy)		1.895 (1.561 to 2.299)^*^ <.001^*^	1.596 (1.301 to 1.959)^*^ <.001^*^	1.593 (1.319 to 1.923)^*^ <.001^*^	
Harms baby (continuous, 1–5, increasing belief)		1.236 (1.078 to 1.417)^*^ .002^*^		1.235 (1.036 to 1.472)^*^ .019^*^	

^1^ *n* = 681 of 906, 75%; 525 missing—classed as making a quit attempt.

^2^ *n* = 199 of 906, 22%; 502 missing—coded as smoking.

^3^OR calculated using Wald’s value for continuous and binary variables and likelihood ratios for categorical variables.

^4^Considered to be statistically significant if *p* < .05

CI = confidence interval; IMD = index of multiple deprivation; OR = odds ratio. ^*^p = <0.05.

In the univariable analyses, five of the smoking-related behaviors were significantly associated with making a quit attempt: a higher percentage reduction in number of cigarettes smoked in early pregnancy (OR = 1.01, 95% CI = 1.01% to 1.01%); a higher nicotine dependence score (HSI) (OR = 0.79, 95% CI = 0.71% to 0.89%); a previous quit attempt of 2–5 weeks (omnibus *p*=.030; <2 weeks OR = 0.83, 95% CI = 0.55% to 1.25%; 2–5 weeks OR = 1.75, 95% CI = 1.03% to 3.09%; 6–11 weeks OR = 1.23, 95% CI = 0.67% to 2.40%; >12 weeks OR = 1.38, 95% CI = 0.92% to 2.05%); higher intention to quit (OR = 1.73, 95% CI = 1.46% to 2.04%); and having set a quit date (OR = 4.80, 95% CI = 1.50% to 15.34%).

All three smoking belief-related predictors significantly predicted making a quit attempt: higher determination to stop (OR = 1.62, 95% CI = 1.40% to 1.88%); higher combined self-efficacy score (OR = 1.90, 95% CI = 1.56% to 2.30%); and higher harm to baby beliefs (OR = 1.24, 95% CI = 1.08% to 1.42%).

In the multivariable analyses, two predictor variables were independently and statistically significantly associated with making a quit attempt; higher intention to quit (OR = 1.50, 95% CI = 1.26% to 1.79%); and higher combined self-efficacy score (OR = 1.60, 95% CI = 1.30% to 1.96%). Being in the MiQuit intervention group was also a statistically significant predictor of quit attempt (OR = 1.53; 95% CI = 1.12% to 2.08%). The variables which were not significantly associated with making a quit attempt in the multivariate analyses were: index of multiple deprivation score; age; partner smoking status; previous pregnancy; length of previous quit attempt; setting a quit date; HSI; strength of urges; change in number of cigarettes in early pregnancy; determination to stop; and beliefs regarding harm to the baby.

### Predictors of Abstinence

In the univariable models, as for the quit attempt outcome, the same five smoking-related behaviors were significantly associated with abstinence: higher percentage reduction in number of cigarettes smoked in early pregnancy (OR = 1.02, 95% CI = 1.01% to 1.02%); higher nicotine dependence score (HSI) (OR = 0.83, 95% CI = 0.75% to 0.94%); longer previous quit attempt (omnibus *p* = <.001; <2 weeks OR = 1.03, 95% CI = 0.60% to 1.78%; 2–5 weeks OR = 1.59, 95% CI = 0.92% to 2.74%; 6–11 weeks OR = 2.028, 95% CI = 1.06% to 3.81%; >12 weeks OR = 2.268, 95% CI = 1.48% to 3.57%); higher intention to quit (OR = 1.37, 95% CI = 1.16% to 1.62%); and having set a quit date (OR = 2.46, 95% CI = 1.49% to 4.04%).

As for quit attempt, all smoking belief-related predictors analyzed were statistically significant predictors of abstinence in univariable analyses: higher determination to stop (OR = 1.47, 95% CI = 1.21% to 1.79%); higher combined self-efficacy (OR = 1.59, 95% CI = 1.32% to 1.92%); and higher harm to baby beliefs (OR = 1.24, 95% CI = 1.04% to 1.47%).

In the multivariable analyses, two variables were independently statistically significantly associated with abstinence: higher percentage reduction in number of cigarettes smoked in early pregnancy (OR = 1.02, 95% CI = 1.01% to 1.02%] *p*=.001); longer previous quit attempt (omnibus *p*=.001; <2 weeks OR = 1.08, 95% CI = 0.63% to 1.87%; 2–5 weeks OR = 1.57, 95% CI = 0.91% to 2.71%; 6–11 weeks OR = 1.95, 95% CI = 1.02% to 3.72%; >12 weeks OR = 2.21, 95% CI = 1.42% to 3.44%). The variables which were not significantly associated with abstinence in the multivariate analyses were: index of multiple deprivation score; age; partner smoking status; previous pregnancy; setting a quit date; intention to quit; HSI; strength of urges; determination to stop; self-efficacy; and beliefs regarding harm to the baby.

### Sensitivity Analyses

In sensitivity analyses using only complete cases, the statistically significant predictor variables from the multivariable analyses remained significant for both the quit attempt and abstinence outcomes. However, additional variables were retained in the models. The percentage change in the number of cigarettes smoked in early pregnancy; the nicotine dependence score (HSI) and previous quit attempts were additional predictors of quit attempts. Combined self-efficacy was an additional predictor of abstinence.

In sensitivity analyses using biochemically validated abstinence in late pregnancy as the outcome, making a previous quit attempt remained a statistically significant predictor (as in the multivariable analyses), but the percentage change in the number of cigarettes smoked in early pregnancy did not retain significance (*p*=.15).

When stratifying by condition, for both quit attempt and abstinence, in the control group, there was no difference from the main results in the predictor variables that were statistically significant. In the intervention group, setting a quit date became statistically significant for both quit attempt (OR = 2.52; 95% CI = 1.22% to 5.22%) and abstinence (OR = 2.46; 95% CI = 1.19% to 5.09%); along with determination to stop in the intervention group for abstinence (OR = 1.50; 95% CI = 1.14% to 1.97%). The variables that were significant in the main results remained significant.

## Discussion

### Key Findings

This study is important as it distinguishes between factors which might influence pregnant smokers attempting to quit smoking, and which might influence successful abstinence, in the same cohort. The results suggested that the factors associated with making a quit attempt differed from those associated with maintaining abstinence. There were significant associations between motivational factors (self-efficacy score and intention to quit) and starting a quit attempt but not with successful abstinence. When considering successful abstinence, the percentage of cigarettes cut down in early pregnancy (<15 weeks) from pre-pregnancy levels was a statistically significant predictor.

### Strengths and Weaknesses

This study has several strengths, including using a large dataset derived from multicenter pooled clinical trials and investigating a broad range of variables. Detailed sensitivity analyses were also carried out using complete cases and biochemically validated outcomes.

However, there are limitations to consider. A proportion of participants (36%) were lost to follow-up across the two studies. For those participants lost to follow-up, it was assumed that the most likely event was that they had made a quit attempt but were still smoking. This is in accordance with the Russell Standard for reporting smoking abstinence.^30^ Aside from the percentage having had a previous pregnancy, those lost to follow-up did not differ from those present at follow-up in the characteristics measured as predictor variables. Complete case analyses, designed to investigate the impact of this attrition rate, revealed additional predictor variables for both quit attempt and smoking abstinence. For maximum statistical power, self-reported abstinence was used as a main outcome variable due to the larger dataset. In sensitivity analyses using biochemically validated abstinence, the percentage of cigarettes cut down in early pregnancy was not a statistically significant predictor of smoking abstinence. Therefore, it is possible that participants who self-reported smoking abstinence may be also more likely to self-report cutting down smoking early in the pregnancy because of perceived social desirability.

It is possible that some participants who would have passed biochemical validation were not included in this because they did not provide a sample. There are limitations of the biochemical validation as it is also possible that some participants could have had a falsely elevated reading, for example due to environmental CO or second-hand smoke exposure. Conversely, false negatives could be due to the accelerated metabolism due to pregnancy or a time-lag between smoking and testing.^32^

We included factors that had been identified as important in previous research,^15^^,^^17^^,^^21^ and the method used to conduct the multivariable analyses examined for potential collinearity between the predictor variables (Pearson’s coefficient <0.8).

As participants volunteered to join the trial, it was possible that they had a higher baseline motivation to quit than the general population of pregnant smokers. However, only 54% of the participants planned to quit in the next month compared to 70% of pregnant smokers in the general population,^33^ suggesting that this was not the case. The MiQuit intervention is designed to be used by pregnant people who are not motivated to quit, as well as those who are; hence, the wide range in participants’ baseline quit motivation.

The study’s inclusion criteria of speaking English fluently, and most of the sample being White British could limit the generalizability of the results. However, UK smoking in pregnancy rates has traditionally appeared highest among those with the White British ethnicity.^9^ Future research should explore whether these results are consistent in other demographics to ensure healthcare intervention equity.

### Findings in Context of Previous Literature

#### Quit Attempt

Having a higher intention to quit and higher combined self-efficacy score were statistically significant predictors of making a quit attempt in this study, which is in line with previous research in the general adult smoking population.^31^^,^^34^ Combined self-efficacy was not statistically significant in pregnant smokers in a previous smaller study,^22^ but the larger dataset and hence higher power in the current study may account for this finding.

Receiving the MiQuit intervention was a statistically significant predictor of quit attempt but not abstinence, which was expected as this is what was reported in the MiQuit trials for the primary outcome.^23^^,^^24^ It was included in this study as a variable to control for any potential effect it may have on the other predictor variables.

#### Smoking Abstinence

A novel variable in our study was “the percentage in number of daily cigarettes cut down in early pregnancy”, which was a statistically significant predictor of smoking abstinence. This measures the change in the number of cigarettes from before pregnancy to baseline (less than 15 weeks pregnant). The multivariable analysis controls for self-reported self-efficacy, intention, and determination, so it does not seem that this is simply because these participants are more self-motivated to reduce their cigarette use and then stop altogether. However, it is important to note that it has previously been reported that women who cut down the number of cigarettes per day may have a compensatory effect as smokers may increase the intensity of the cigarettes they smoke, and so their exposure to products of combustion may not necessarily be lower.^34^

A longer previous quit attempt (over 6 weeks) was a statistically significant predictor of abstinence, but not of quit attempt. This may be because women who have had a previous long quit attempt have learnt from this attempt about what works well for them. In the Emery et al. study, previous quit attempt was not a statistically significant predictor, this may have been because it was categorized differently, as: “any quit attempt prior to baseline” and “duration of longest quit attempt prior to baseline”, or due to the higher statistical power of this study.

It should be noted that although women were more likely to make a quit attempt, having high intention to quit or self-efficacy was not an independent predictor of being more likely to be successful with smoking abstinence. A previous study had suggested that scoring highly on motivational factors for smoking abstinence could even negatively impact the chance of smoking cessation, which may be because participants may think that their high motivation is sufficient for quitting and may engage less with additional cessation interventions (eg, nicotine replacement therapy (NRT)).^34^

Setting a quit date is an evidence-based feature of successful smoking cessation programs for pregnant women,^35^ and National Centre for Smoking Cessation and Training (NCSCT) guidance^36^ for stopping smoking in pregnancy is to set a quit date as soon as possible and structure follow-up around it. It was a significant predictor of both quit attempt and abstinence only in the intervention group. This suggests that setting a quit date without formal cessation support may be less likely to lead to abstinence than when accompanied by an intervention, although this needs to be confirmed by future research.

The smoking status of the partners of participants (categorized into partner non-smoker, single, and partner smoker) was not shown to be a statistically significant predictor of either quit attempt or abstinence in this study. Previous smaller studies have shown that having a partner who smokes reduces the chance of smoking cessation.^19^^,^^37^ Previous research has also identified HSI as a statistically significant predictor of pregnancy smoking cessation^17^^,^^22^; this was the only significant predictor in multivariate models in Emery et al. HSI may not have appeared significant in this study because of undetected collinearity with the percentage change in cigarettes smoked in early pregnancy (Pearson’s coefficient −0.442, *p* < .001), which was not considered in previous studies.^17^

In this study, follow-up was at 36 weeks’ gestation; other studies have further considered post-partum relapse after smoking cessation during pregnancy, which occurs in around 43% of women, and is influenced by maternal age, having a partner who smokes, and the self-reported likelihood of postpartum relapse.^11^

### Implications for Smoking Cessation Interventions

Where smoking cessation support is routinely offered in pregnancy, this often accompanies antenatal care, which often starts at 10 weeks’ gestation or later, with both the United Kingdom and Australia having adopted this model.^38^^,^^39^ Following this initial assessment, pregnant smokers are referred to stop smoking services, where they are supported with advice with a goal to quit smoking, and the main effective interventions used are face-to-face behavioral support and nicotine replacement therapy.^40^ Importantly, this study suggests that women who cut down the number of cigarettes that they smoke early in pregnancy are more likely to stop smoking. Therefore, targeting support for smoking cessation earlier in pregnancy, or as part of pre-conceptual planning, could reduce the number of women who continue to smoke throughout pregnancy, and thus improve outcomes for both mother and baby.

However, in those women who have unplanned pregnancy, preconceptual counseling would not be possible. Women who have an unplanned pregnancy are more likely to continue smoking during pregnancy.^41^ Unplanned pregnancy is more common in women of lower socioeconomic status,^42^ and thus it would be important that, along with increasing pre-conceptual smoking cessation interventions, addressing smoking earlier in pregnancy and in women of childbearing age is also a priority.

## Conclusions

In conclusion, results suggest that motivational factors including intending to quit and being more confident of success help explain the initiation of quit attempts but not successful cessation. The latter is more likely if women report reduced smoking in early pregnancy or of previous smoking abstinence. To assist women with starting quit attempts, and then become smokefree within them, health professionals may require quite different support strategies.

These findings suggest that targeting smoking cessation interventions earlier in pregnancy or as part of pre-conceptual planning^43^ may have a positive impact on the number of women who successfully achieve cessation during pregnancy. It would be important, however, to ensure not to exacerbate any socioeconomic disparity due to unplanned pregnancies, by making pregnancy-specific smoking cessation services easily accessible to all women in early pregnancy.

## Supplementary Material

Table_1_-_for_submission_ntaf262

Table_2_-_for_submission_ntaf262

Supplementary_information_-_for_submission_ntaf262

## Data Availability

Data not publicly available.
