# Supplementary material for: Predictors of Quit Attempts and Successful Smoking Abstinence in Pregnancy: An Exploratory Analysis of a Pooled Clinical Trial Dataset
Source: Nicotine Tob Res. 2025 Dec 24;28(10):1735–43. doi: 10.1093/ntr/ntaf262 (PMC13601959; doi:10.1093/ntr/ntaf262)
Supplement: Supplementary file 1 [file supplementary_information_-_for_submission_ntaf262.docx]

**SUPPLEMENTARY INFORMATION**

| **Appendix 1: Baseline Characteristics of MiQuit Pilot and MiQuit3 Trials** |  |
| --- | --- |

|  | **MiQuit Pilot** | | **MiQuit3** | |  |
| --- | --- | --- | --- | --- | --- |
|  | **N=407^[1]^** | **%** | **N=1002^a^** | **%** | **p value ^[2]^** |
| **Trail Details** | | | | | |
| **Demographics** | | | | | |
| **IMD Rank** (*median, interquartile range*)^[3]^ | 5768.00,  9903 | | 6923.00  11216 | | 0.145^[4]^ |
| *N, missing data* | *399, 8* | | *989, 13* | |  |
| **Age** | | | | | |
| Age (*mean, standard deviation)* | 26.50,  5.665 | | 27.38,  5.676 | | 0.015* ^[5]^ |
| **Smoking status of partner** | | | | | |
| Partner smokes | 263 | 64.6% | 643 | 64.2% | 0.984^[6]^ |
| Partner doesn’t smoke | 78 | 19.2% | 193 | 19.3% |  |
| No partner | 66 | 16.2% | 166 | 16.6% |  |
| **Ethnicity^[7]^** | | | | | |
| White | 373 | 91.9% | 945 | 94.7% | 0.63^6^ |
| Mixed race | 17 | 4.2% | 34 | 3.4% |  |
| Other | 16 | 3.9% | 19 | 1.9% |  |
| *N, missing data* | *406, 1* | | *998, 4* | |  |
| **Smoking Behaviours** | | | | | |
| **Percentage change in number of cigarettes smoked in early pregnancy^[8]^** | | | | | |
| Percentage change in number of cigarettes smoked in early pregnancy  (*Median, Interquartile range)* | 50.00,  42 | | 50.00,  33 | | 0.034* ^4^ |
| **Nicotine dependence (Using HSI^[9]^)** | | | | | |
| Nicotine dependence (Using HSI) (*Mean, standard deviation)* | 1.97,  1.388 | | 1.96,  1.362 | | 0.882^5^ |
|  |  | |  | |  |
| **Longest quit attempt prior to baseline** | | | | | |
| Not attempted | 94 | 23.1% | 239 | 23.9% | 0.846^6^ |
| <2 weeks | 95 | 23.3% | 207 | 20.7% |  |
| 2-5 weeks | 58 | 14.3% | 143 | 14.3% |  |
| 6-11 weeks | 30 | 7.4% | 73 | 7.3% |  |
| >12 weeks | 130 | 31.9% | 340 | 33.9% |  |
| **Previous pregnancy (proxy for smoking in previous pregnancy)^[10]^** | | | | | |
| Yes | 276 | 67.8% | 663 | 66.2% | 0.553^6^ |
| No | 131 | 32.2% | 339 | 33.8% |  |
| **Strength of smoking urges (scale 1= no urges; 6= extremely strong)** | | | | | |
| Strength of smoking urges *(mean, standard deviation)* | 3.09,  1.073 | | 3.13,  0.993 | | 0.474^5^ |
| *N, missing data* | *407, 0* | | *963, 39* | |  |
| **Intention to quit smoking** | | | | | |
| Within 2 weeks | 132 | 32.4% | 253 | 25.3% | 0.006* ^6^ |
| Within 30 days | 114 | 28.0% | 258 | 25.8% |  |
| Within 3 months | 125 | 30.7% | 398 | 39.8% |  |
| No intention to quit | 36 | 8.8% | 90 | 9.0% |  |
| *N, missing data* | *407, 0* | | *999, 3* | |  |
| **Set a quit date** | | | | | |
| Yes | 22 | 5.4% | 385 | 94.6% | 0.574^6^ |
| No | 62 | 6.2% | 940 | 93.8% |  |
| **Smoking Beliefs ^[11]^** | | | | | |
| Determination to stop smoking for remainder of pregnancy (median, interquartile range) | 4.00,  2 | | 4.00,  1 | | 0.005* ^4^ |
| Combined self-efficacy^[12]^ (*mean, standard deviation*) | 2.70,  0.799 | | 2.68,  0.848 | | 0.986^5^ |
| Beliefs regarding harm to baby (*median, interquartile range)* | 5.00,  2 | | 5.00,  1 | | 0.010* ^4^ |
| *N, missing data* | *407, 0* | | *1001, 1* | |  |

1. N=407 for MiQuit Pilot, N=1002 for MiQuit 3, unless otherwise stated
2. Tested using chi-squared test (frequencies) or t test (means), for normally distributed or Mann Whitney U (medians) for non-parametric data; two-tailed.
3. Index of Multiple Deprivation rank (Based on Postcode based on 2015 data for both MiQuit3 and MiQuit Pilot). MD Rank in England covers domains of income, employment, health, education, crime, access to services and living environment.
4. Mann-Whitney U Test
5. T test
6. Chi squared test
7. Ethnicities with percentage >1% will be included in the table, and in those ethnicities where there is less than 1% of the participants in that category, will be grouped as ‘other’.
8. Percentage change in cigarettes smoked in early pregnancy, calculated using reported number of cigarettes smoked prior to pregnancy vs reported smoked at baseline.
9. Using Heaviness of Smoking Index (HSI) score; combining the score of two items: cigarettes per day (1–5 = score of 0, 6–10 = 1, 11–20 = 2, 21–30 = 3, >30 = 4) and time to first cigarette after waking (>2h = 0, 1–2h = 1, 31–59min = 2, ≤30min = 3). A combined score of 0–2 = low dependence, 3–4 = medium dependence, 5–7 = high dependence.
10. Previous studies have shown that smoking in a previous pregnancy and having had a previous pregnancy are very closely related in a similar cohort.
11. Smoking beliefs questions were answered using a scale where 1- not at all; 2- a little; 3- moderately; 4-very much; 5 extremely.
12. The average of four items measuring confidence to avoid smoking: one for the remainder of the pregnancy, and three in different types of tempting situation (after a meal; with other smokers; and when anxious/stressed), as used in previous studies (α = 0.81).
