# Supplementary material for: Predictors of Quit Attempts and Successful Smoking Abstinence in Pregnancy: An Exploratory Analysis of a Pooled Clinical Trial Dataset
Source: Nicotine Tob Res. 2025 Dec 24;28(10):1735–43. doi: 10.1093/ntr/ntaf262 (PMC13601959; doi:10.1093/ntr/ntaf262)
Supplement: Supplementary file 3 [file table_2_-_for_submission_ntaf262.docx]

**Table 2. Univariate & Multivariate Predictors of Making a Quit Attempt and Predictors of Self-reported Abstinence in Pregnant Smokers**

|  | | **Quit Attempt^[1]^** | | **Self-reported abstinence^[2]^** | |
| --- | --- | --- | --- | --- | --- |
| **Predictor Variable** | | **Univariate *OR^[3]^ (95% CI^[4]^)***  ***P Value^[5]^**** | **Multivariate OR (95% CI)**  **P value*** | **Univariate *OR (95% CI)***  ***P Value**** | **Multivariate OR (95% CI)**  **P value*** |
| **Condition** (0=usual care, 1=MiQuit) | | 1.535 (1.135 -2.075)*  0.005* | 1.527 (1.120- 2.082)*  0.008* | 1.246 (0.917 – 1.693)  0.160 |  |
| **IMD^[6]^ Score** (continuous, 15-32742, increasing IMD) | | 1.000(1.000-1.000)  0.237 |  | 1.000 (1.000 – 1.000)  0.475 |  |
| **Age** (continuous, 16-43, increasing age) | | 0.982 (0.957-1.008)  0.169 |  | 1.017 (0.990 – 1.044)  0.221 |  |
| **Partner smoking status**  (Reference= Partner non-smoker) | **Overall likelihood ratio** | LR Chi squared = 3.223  DF = 3  p=0.200  *(In STATA LR Chi^2^ = 3.22; P = 0.1996)* |  | LR Chi squared 2.698  DF = 2  p=0.259 |  |
|  | **Category** | | | | |
|  | *Single* | 0.678 (0.401 – 1.138)  0.095 |  | 0.785 (0.476 – 1.280)  0.335 |  |
|  | *Partner smoker* | 0.699 (0.452-1.050)  0.881 |  | 0.729 (0.505 – 1.064)  0.096 |  |
| **Percentage change number of cigarettes early pregnancy**  (Continuous, -300- 97%, increasing percentage cut down) | | 1.009 (1.005-1.013)*  <0.001* |  | 1.016 (1.010 – 1.023)*  <0.001* | 1.015 (1.009 – 1.022)*  0.001* |
| **Nicotine dependence score** (continuous, increasing HSI) | | 0.794 (0.711-0.887)*  <0.001* |  | 0.834 (0.745- 0.935)*  0.002* |  |
| **Previous quit attempt**  (Reference = Quit not attempted) | **Overall LR** | LR Chi^2^= 10.70  p= 0.030* |  | LR Chi^2^= 20.987  p= <0.001 | LR Chi^2^= 17.99 p= 0.001* |
|  | **Category** | | | | |
|  | *<2 weeks* | 0.832 (0.551-1.254)  0.379 |  | 1.032 (0.597- 1.777)  0.909 | 1.084 (0.627 – 1.874)  0.773 |
|  | *2-5 weeks* | 1.752 (1.030 – 3.087)  0.044 |  | 1.589 (0.923 – 2.735)  0.093 | 1.568 (0.908 – 2.707)  0.106 |
|  | *6-11 weeks* | 1.230 (0.669 – 2.399)  0.522 |  | 2.028 (1.056 – 3.808)  0.030* | 1.949 (1.023 – 3.716)*  0.043* |
|  | *>12 weeks* | 1.375 (0.920-2.053)  0.119 |  | 2.268 (1.476 – 3.567)*  <0.001* | 2.208 (1.416 – 3.444)*  <0.001* |
| **Previous pregnancy** (0= No previous pregnancy; 1=Had previous pregnancy) | | 0.793 (0.572 – 1.097)  0.162 |  | 0.759 (0.555 – 1.039)  0.085 |  |
| **Strength of urges** (continuous, 1-6, increasing strength) | | 0.952 (0.823-1.102)  0.509 |  | 0.941 (0.807 – 1.097)  0.439 |  |
| **Intention to quit** (continuous, 1-4, increasing intention) | | 1.726 (1.463 – 2.036)*  <0.001* | 1.501 (1.261 – 1.788)*  <0.001* | 1.369 (1.159 – 1.616)*  <0.001* |  |
| **Set a quit date** (0=no, 1=yes) | | 4.800 (1.502- 15.344)*  0.008* |  | 2.457 (1.492 – 4.044)  <0.001* |  |
| **Determination to stop** (continuous, 1-5, increasing determination) | | 1.623 (1.399-1.882)*  0.001* |  | 1.471 (1.212 – 1.786)*  <0.001* |  |
| **Combined self-efficacy** (continuous, 1-5, increasing self-efficacy) | | 1.895 (1.561-2.299)*  <0.001* | 1.596 (1.301 – 1.959)*  <0.001* | 1.593 (1.319 – 1.923)*  <0.001* |  |
| **Harms baby** (continuous, 1-5, increasing belief) | | 1.236 (1.078-1.417)*  0.002* |  | 1.235 (1.036 – 1.472)*  0.019* |  |

1. n= 681 of 906, 75%; 525 missing – classed as making a quit attempt
2. n=199 of 906, 22%; 502 missing – coded as smoking
3. *OR = Odds ratio. OR calculated using Wald’s value for continuous and binary variables and likelihood ratios for categorical variables.*
4. *CI= Confidence interval*
5. *Considered to be statistically significant if p<0.05*
6. *IMD = Index of Multiple Deprivation*
